# Family caregiver involvement and role in hospital at home for adults: the patients’ and family caregivers’ perspective - a Norwegian qualitative study

**DOI:** 10.1186/s12913-023-09531-3

**Published:** 2023-05-17

**Authors:** Lillian Karlsen, Bente Prytz Mjølstad, Bjarte Bye Løfaldli, Anne-Sofie Helvik

**Affiliations:** 1 The Centre for Health Innovation, Øvre Enggate 8B, Kristiansund N, N-6509 Norway; 2 Faculty of Medicine and Health Sciences, Department of Public Health and Nursing, Norwegian University of Science and Technology, Postboks 8905, Trondheim, N-7491 Norway; 3 General Practice Research Unit, Department of Public Health and Nursing, Norwegian University of Science and Technology, Postboks 8905, N-7491 Trondheim, Norway

**Keywords:** Home care services, Hospital-based, Hospital at home, HAH, Family caregiving, Involvement and role, Qualitative research

## Abstract

**Background:**

Hospital at home (HaH) provides acute healthcare services in patients’ homes instead of traditional in-patient care. Research has reported positive outcomes for patients and reduced costs. Although HaH has developed into a global concept, we have little knowledge about the involvement and role of family caregivers (FCs) of adults. The aim of this study was to explore FC involvement and role during HaH treatment as perceived by patients and FCs in a Norwegian healthcare context.

**Methods:**

A qualitative study was carried out among seven patients and nine FCs in Mid-Norway. The data was obtained through fifteen semi-structured interviews; fourteen were performed individually and one as duad interview. The age of the participants varied between 31 and 73 years, and mean age of 57 years. A hermeneutic phenomenological approach was used, and the analysis was performed according to Kvale and Brinkmann’s description of interpretation.

**Results:**

We identified three main categories and seven subcategories regarding FC involvement and role in HaH: (1) Preparing for something new and unfamiliar, including the subcategories `Lack of involvement in the decision process` and `Information overload affecting caregiver readiness`, (2) Adjusting to a new everyday life at home, including the subcategories `The critical first days at home`, `Coherent care and support in a novel situation`, and `Prior established family roles influencing the new everyday life at home`, (3) FCs` role gradually diminishes and looking back, including the subcategories `A smooth transition to life beyond hospital at home` and `Finding meaning and motivation in providing care`.

**Conclusions:**

FCs played an important role in HaH, although their tasks, involvement and effort varied across different phases during HaH treatment. The study findings contribute to a greater understanding of the dynamic nature of the caregiver experiences during HaH treatment, which can guide healthcare professionals on how they can provide timely and appropriate support to FCs in HaH over time. Such knowledge is important to decrease the risk of caregiver distress during HaH treatment. Further work, such as longitudinal studies, should be done to examine the course of caregiving in HaH over time to correct or support the phases described in this study.

**Supplementary Information:**

The online version contains supplementary material available at 10.1186/s12913-023-09531-3.

## Background

In the last decades, hospital at home (HaH) has developed into a global concept, in accordance with policy shift emphasizing bringing healthcare closer to patients’ homes [1, 2]. HaH provides acute healthcare services in patients’ homes for a broad range of conditions that otherwise would require in-patient care and is always offered for a limited time [2, 3]. There is a wide range of models for the HaH concept, in which the interventions, population, service structure and program components vary [4]. The HaH models also vary in the degree the service replaces in-patient treatment [5].

In Norway, HaH was established in 2008 as a healthcare service for children and adolescent [6]. Since 2019, outreaching hospitals and HaH have become a part of the Norwegian government’s strategy to achieve sustainable and patient-centered healthcare services [7]. Thus, also the number of HaH for adults have started to grow. In Norway, the healthcare services is a public responsibility and the formal healthcare services are primarily organized in two levels, the specialist healthcare services (the hospitals) and the primary healthcare services (the municipalities) [8]. The most prevalent HaH model in Norway is the one where treatment and care is offered in patient’s home by hospital healthcare professionals (HHP) under supervision of a hospital physician who has the medical responsibility for the patient [9]. However, an alternative model has been developed, where municipal healthcare professionals (MHP) replace HHP providing care in patient’s homes, but still under supervision of the hospital physician [9]. The latter model corresponds to the Norwegian health policy objectives aiming for patients to receive cohesive and coordinated services across hospitals and municipalities, and to minimize geographical inequalities [7].

Research suggests that HaH may be an acceptable solution for the increased need for care conflicting with limited hospital resources and lack of hospital beds due to demographic changes in the population [5, 10]. Outbreaks of pandemics, such as COVID-19, have further highlighted the benefits of HaH [11]. HaH have been shown to improve patients’ quality of life [12] and satisfaction [3, 13], reduce readmissions to the hospital [3, 14], and contribute to lower mortality [3, 15].

Family caregivers (FCs) constitute a major resource in today’s healthcare systems [16], estimated to account for approximately 60% of the total care needed in EU-countries [17]. In Norway, family caregiving is estimated to be approximately equal to the efforts in the formal healthcare services provided by the municipalities [18, 19]. The need for FCs is expected to increase as the populations get older, but the number of disposable FCs is expected to decline due to changes in demographics [20].

Furthermore, FCs face more physical and mental health challenges than non-caregivers [21], and caregiving is linked to increased risk of mortality [22]. However, research also shows an improvement in psychological health [23] and longevity [24] in some FCs. It is important to know how FCs are involved and what role they play in various healthcare models to target adequate professional support to FCs with the greatest need [25]. The caregiver role is suggested to be dynamic [26] and thus, there is a need to identify and understand a point in time at which FCs will need support [27].

Even so, we know little about experiences of FC involvement and role in the HaH context. Previous qualitative research has mostly focused on satisfaction with HaH [28, 29], the overall HaH experience [30, 31], and identification of drivers and barriers to implementing and improving HaH [32, 33]. These studies describe lack of FC involvement in the decision making for choosing HaH [33], and suggest that caregiving in HaH affect the patient-FC relationship [30, 34]. Furthermore, the experiences with FC involvement and role are described differently, possibly linked to the HaH model used. HaH models characterized by complex treatment and severe illness are associated with higher caregiver burden [33].

To our knowledge, no study has, as its main objective, explored the experiences of involvement and role of FCs in HaH for adults. Furthermore, we have not found any study investigating HaH for adults in Norway. The aim of this study was to explore FC involvement and role during HaH treatment as perceived by patients and FCs in a Norwegian healthcare context.

## Methods

We used a hermeneutic phenomenological approach and carried out a qualitative study based on individual interviews and one dual interview with FCs and patients. A qualitative approach with interviews was appropriate as we aimed to explore how individuals experience a phenomenon or event that has already occurred [35].

### Study setting

This study was conducted in a healthcare area in Mid-Norway with approximately 50.000 inhabitants. The HaH model patients were a part of, and FCs were involved in, is presented in Table 1.

**Table 1:** Characteristics of the HaH model in Mid-Norway

**Elements of the HaH model in Mid-Norway** - Is partly a substitute for in-patient treatment (Early discharge) - A hospital physician has the overall medical responsibility and develops an individual plan for medical treatment and tailored care at home - The hospital pharmacist prepares and supplies medicine and equipment to patients’ homes - Municipal healthcare professionals contribute with administrating intravenous antibiotic in infusion pump, observations and monitoring of patients at home - Patients have a digital safety alarm which allows communication with a response center 24/7 - Video consultations enable remote care	**Available for HaH treatment** - Patients over 18 years of age with the capacity to consent - Patients with chosen medical, post-surgical, or neurological diagnosis in a stable condition that need prolonged hospital-monitored iv antibiotic treatment - Patients motivated and willing to receive treatment in HaH - The home must be suitable (internet-access, hygienic standard)

### Sample

We included both the patient and FC perspective to broaden our understanding of the caregiving experience, since caregiving is a relational process [36] and this approach may provide a more comprehensive picture of the caregiving phenomenon than interviewing only FCs [37]. Patients who had been included in HaH treatment in Mid-Norway during the last two years (2019–2021), including the COVID-19 pandemic period, and their FC were actual for the study. The participants were recruited with assistance from the administration for the hospital and the municipal healthcare services. We used a purposeful sampling strategy, including participants representing a variation in age and gender, diagnosis, and length of stay in HaH [38]. When no new relevant information was forthcoming from the interviews, the authors decided to stop recruiting participants [39]. The authors did not establish any relationship with the study participants prior to the interviews. One patient and one FC did not want to participate in the study.

### Data collection and interview guide

The interview guide was developed by the authors based on their knowledge of the field, both theoretically and from clinical experience. A pilot study with the FC representative in the study led to some adjustments of the interview guide. The main themes addressed the informants’ experiences regarding FC involvement and role during HaH treatment, including how everyday life were affected. The interview guide is added as an additional file (Additional file 1). The questions were open ended and the order flexible, allowing the interviewer to pursue new and important topics brought up by the participants [40]. The interviewer sought to build a trustful relationship with the participants and emphasized checking out interpretations during the interviews [41]. Before the interviews started, the participants were informed about the interviewer’s background as a former nurse and as a researcher interested in understanding FC involvement and role in different care contexts.

Out of 15 conducted interviews, 14 were individual while one was a duad interview at the request of the patient/FC. The interviews were conducted where the study participants found it most suitable: ten interviews were carried out in the participants’ homes, two interviews in a neutral office and three interviews were performed as telephone interviews. The first author (LK) conducted all the interviews, guided by the semi-structured guide. The interviews lasted between 15 and 68 min, with an average length of 40 min. Each interview was digitally recorded and transcribed verbatim by the first author (LK) and by an external transcriber. The interviewer wrote field notes after the interview, including her immediate reflections. The transcripts were not returned to the participants for comments or corrections and no repeat interviews were carried out.

### Analysis and interpretation of interview data

The analysis followed Kvale and Brinkmanns three-level interpretation: (1) self-understanding, (2) general understanding and common sense, and (3) final interpretation based on theoretical understanding [42].

In the first level a descriptive analysis of the transcribed interviews was conducted. The transcribed texts were condensed mainly by first author (LK) identifying, rephrasing and at last elucidating natural meaning units in the text based on the purpose of the study, aiming to identify the meaning content. Author ASH and BPM were involved in this process to ensure that the meaning condensation represented a rephrased condensation of the meaning of the interviewee`s statements from their own viewpoints, as understood by the first author (LK). The second level of interpretation was based on critical common sense understanding, where general knowledge was added in the authors` (LK, ASH, BPM, BBL) discussions to uncover nuanced meanings as perceived by all authors and to amplify and further develop preliminary subcategories. In Table 2, we show examples of how the natural units of meaning from the transcribed interviews were condensed by the authors and labeled with preliminary subcategories. In the third level, these preliminary subcategories were enriched by existing research and relevant theories to deepen and broaden the authors` understanding of the data. Patterns and connections within the preliminary subcategories were linked together to the final categories and split into main categories and subcategories.

**Table 2:** Examples from the process of meaning condensation

Unit of meaning	Meaning condensation	Preliminary subcategories
“While it was going on, the relationship between us became different. Because now it was me who had the household. I had not been used to that. And it was a challenge, for my wife and for me. To deal with the reaction that I didn’t do it the same way”	The relationship between patient and family caregiver changed during the hospital at home period. It became challenging for both that he carried out his new tasks differently from what his spouse had used and done.	Hospital at home affecting the relationship
“In the beginning I was thinking about what would possibly happen if she got any reactions to the medicine. I thought that it was possible to get sick of it. It did not happen, of course. And the nurses were ready if we needed help. It did not happen. But the thought of it, that if she got a reaction or something (.)”	The family caregiver was stressed out at the first period at home because thinking about the risks	The critical first phase

First author (LK) had the main responsibility for the analysis but collaborated with the rest of the research team (ASH, BPM and BBL) throughout the analysis and interpretation process. All authors contributed to discussions of the main results. No software was used to manage the data in this study. Preliminary subcategories were also presented for and discussed with the FC representative in the study.

### Ethical considerations

The research project was exempt from formal review by the Regional Committee for Medical and Health Research Ethics in Norway (ref. no. 267185) as the purpose of the research project was not to generate new knowledge about health and disease. The research project was registered and conducted in accordance with the protocol of the Norwegian Centre for Research Data (ref. no. 183099). The participants were given both verbal and written information about the study and those included gave a written consent to participate in the study prior the interviews.

### Pre-understanding

Our pre-understanding as a research team was characterized by our various professional background as well as scientific experience; two nurses, one GP and one neuroscientist with background from research, development, and innovation. The first author is a PhD-student and ASH, BMP and BBL are experienced researchers. The first author (LK) and BBL had previous knowledge about the HaH model in Mid-Norway. Thus, the interpretations we present are influenced by a broad range of experiences and perceptions to the phenomenon of FC involvement and role in HaH.

The consolidated criteria for reporting qualitative studies, COREQ, was used to ensure comprehensive reporting [43]. This check list is added as an additional file (Additional file 2).

## Results

Sixteen informants participated in the study. The average duration HaH was 26 days, within a range of 14–49 days. Further characteristics of the study participants are presented in Table 3.

**Table 3:** Characteristics of the study participants

Family caregiver (FC) (n = 9)				Patient (n = 7)		
Participant	Age	Gender	Relationship of FC to patient	Participant	Age	Gender
FC1	65–75	M	P1s husband	P1	55–65	F
FC2	65–75	M	P2s husband	P2	65–75	F
FC3*	25–35	F	Other relationship to P2	P2	-	-
FC4	65–75	M	P3s husband	P3	65–75	F
FC5**	45–55	F	P4s wife	P4	55–65	M
FC6	55–65	F	P5s wife	P5	65–75	M
FC7***	35–45	F	Other relationship	-	-	-
FC8	55–65	M	P6s husband	P6	55–65	F
FC9	65–75	F	P7s wife	P7	65–75	M

FC = family caregiver, P = patient, F = female, M = male

*Not living in the same house as the patient (the other participants in the study were living together)

**Several family members are living in the household

***The patient did not want to participate in the study

Based on the patients’ and FCs’ experiences we identified three main categories regarding FC involvement and role during HaH: (1) Preparing for something new and unfamiliar, (2) Adjusting to a new everyday life at home, (3) FCs’ role gradually diminishes and looking back. Thematic subcategories are covering various aspects of the main categories (Table 4).

**Table 4:** Overview of the main categories and subcategories

Main categories	Subcategories
Preparing for something new and unfamiliar	Lack of involvement in the decision process
	Information overload affecting caregiver readiness
Adjusting to a new everyday life at home	The critical first days at home
	Coherent care and support in a novel situation
	Prior established family roles influencing the new everyday life at home
FC’s role gradually diminishes and looking back	A smooth transition to life beyond hospital at home
	Finding meaning and motivation in providing care

## Preparing for something new and unfamiliar

### Lack of involvement in the decision process

Overall, the FCs were left out of the decision process of choosing HaH instead of in-patient treatment. The decision was usually made by the patients and the attending physicians at the hospital. FCs were subsequently informed by the patients.

The decision for HaH was influenced by the patients’ own wishes to come home instead of being hospitalized for several weeks. Some patients stated that at this point they did not think of their family members much, nor of the FCs responsibilities. One patient stated:

“I thought mostly about myself and coming home. I was selfish. It was just me who mattered”.(P2)

In some cases, the patient’s eagerness to return home led to hasty transition from hospital to home.

FCs mainly expressed understanding for the patients wishes and decisions to enter the HaH treatment, also when not being consulted in the first place. One FC expressed his thoughts when his wife told him about her decision of entering HaH:

“As I said then, I have been used to take things as they come (.) when she herself was pleased to get out of the hospital then it was fine by me”. (FC1)

Hence, being at the hospital, before returning home, FCs did not normally question their family member’s decision of entering HaH. However, at the point of transfer from hospital to home several FCs described ambivalence toward the homecoming. Although FCs looked forward to the patient’s return home and being together as a family again, they struggled with their own worries about what was to come. One FC described such ambivalence when it was clear that her husband would soon return home:

“He was so excited about HaH, so they organized it quickly. He came home after a few days after the decision. At this time, I thought; *should you come home now?* Because he was still weak and needed a lot of equipment. At the same time, he was an adult who wanted to go home. So, I thought; *you are not sick, and we have a short way to the hospital if anything happens”.* (FC5)

### Information overload affecting caregiver readiness

Both patients and FCs emphasized the importance of FC involvement when receiving information and training before the homecoming. However, many also experienced being overwhelmed by the information.

FCs generally reported that they were more capable to help patients learn and remember important information and procedures when being well informed. One patient described why she needed help to remember:

“You are not quite yourself when you are in the hospital. Maybe you are a little confused when you get home too”. (P1)

One FC said that the training she received at the hospital, helped her to support her husband in handling the equipment and procedures correctly:

“I was engaged in all the training before the transfer from hospital to home. My husband changed the hose himself when we came home, but he wanted me to be there and help him to remember so everything was done right”. (FC5)

The needs for information and training varied. Several FCs experienced that the information and training they received were comprehensive, including many details related to technical equipment and procedures. For some FCs this was overwhelming, and they described it as difficult to sort out the most essential information they needed to be ready for the caregiver role. The comment below illustrates that the feeling of being overwhelmed by information could contribute to FC distress:

“The healthcare professionals at the hospital showed us everything. And then I got a little stressed. Are we supposed to deal with all this at home? And then we said to them; maybe you do not have to tell us everything?”. (FC5)

A few FCs did not want to take part in the training or receive information about HaH before transfer to home. This was typical for FCs of patients who had a professional healthcare background. These FCs trusted that the patient him/herself was best able to receive the information and training, and that the patient would convey the information they needed after homecoming. One FC commented that she was reluctant to participate in the training. She found it natural that her husband took the main responsibility since he was a healthcare professional. Her husband confirmed this:

She thought I could manage all of it myself. I have my background as a healthcare professional right? And I liked to be in control. So, I took all the training myself”. (P5)

## Adjusting to a new everyday life at home

### The critical first days at home

Overall, the FCs described how they were especially stressed during the first days at home due to several reasons.

One FC expressed that she worried about possible complications in relation to the intravenous antibiotic treatment:

“In the beginning I was thinking about what could happen if she got any reactions to the medicine. I thought that it was possible to get sick from it. It did not happen, of course. And the municipal healthcare professionals were ready if we needed help. It did not happen. But the thought of it, if she got a reaction or something (.)”. (FC3)

Another FC voiced concerns related to potential deterioration of her husband’s state of health after returning home. She had previously experienced her spouse to underestimate his state of health and symptoms, thus she did not trust what he reported to her:

“When he got this infection, he got really ill. He was admitted to the hospital with remarkably high infection parameters. The night before, when I wanted to call the emergency, he would not let me. No, no, he was not ill. No, do not bother the health services”. (FC5)

The patients in the study told they sensed this FC stress and worries in the beginning of the HaH stay. Some patients also described they were affected by this initial FC stress. One patient described that her husband was watching her closely during the first period at home, and that his worries stressed her as well:

“In the beginning he was on guard. He was watching me all the time, asking me: are you alright? Have you eaten? It made me nervous. It was a bit too much of a good thing”. (P3)

Also, most FCs initially felt stressed by the medical equipment, especially the infusion pump. As one FC put it:

“There were some small problems with that pump (.) the alarm went off. To begin with we were of course on the alert, high up”. (FC4)

The role and responsibilities of FCs in administrating the infusion pump and handling procedures and care of the central venous catheter varied.

### Coherent care and support in a novel situation

Continuity of care and support from MHPs was emphasized by both FCs and patients. FCs trusted these professionals and felt safe when sharing the caregiver responsibility with them. The patients appreciated MHPs support of the FCs, as they themselves said, they did not have the capacity for it.

Several FCs embraced the regular home visits from the MHP. For example, one FC described these visits as supportive as it gave a feeling of shared responsibility:

“The visits of the professionals were just a break in everyday life, and I did not see their visits as a burden at all (.) nor the other way, it was reassuring. So, I said that having someone else to blame, in a way”. (FC1)

Another FC stated she felt safe because of the availability and response of the MHP, also between their regular visits:

“If we needed help, we called them. We had a separate phone number for these healthcare professionals. And if we called, they were here right away. Quite simply. It was (.) you hung up, and five minutes after they were here. I felt that was safe”. (FC6)

Furthermore, FCs emphasized the stability and continuity within the MHP team. FCs mostly met the same professionals over time and experienced that these professionals had in depth knowledge of both the patient and the family situation at home. One FC reported high satisfaction with the MHP, and that the continuity of care was leading to high-quality care and trust towards these professionals. It was expressed like this:

“The professionals who visited us at home were amazing. We became quite well acquainted with them because it was the same nurses who came back. They were superb to me too. They sorted it all out. It was reassuring for both my wife and me. We had a particularly good relationship. I felt like they knew what they were doing, that they knew their job “. (FC4)

Patients also acknowledged the support for FCs. They described it as important for them as patients to know that the FC felt safe and comfortable in the situation. One patient reflected:

“It was very important for me that the nurses who visited us at home cared for my wife too, not only me as a patient. I think it is important that they ask the other part how they feel about being a caregiver at home. I didn’t think clearly about her needs at that time, I was too sick”. (P5)

### Prior established family roles influencing the new everyday life at home

Prior roles and relationship within the family seemed to influence FC involvement in HaH and how the FCs adapted to the new situation at home. Also, the relationship between patients and FCs seemed to be influenced during HaH, both in good and less good ways.

FCs reflected how both HHP as well as the patients themselves seemed to overestimate the patient’s capacity of self-care if he or she had a professional healthcare background. One FC explained:

“My husband was very keen to get it done, so he said we will sort this out. But maybe they believed in him too much, due to him being a professional. He is good at his work but treating oneself is not the same. Healthcare professionals at the hospital should maybe have been more critical of whether he was able to manage everything himself“. (FC9)

FCs of such patients seemed less prepared before homecoming, still they had to take a great role in assisting the patient at home. The comment below illustrates how feelings of expectations from and dependency on the patient invoked frustration in the FC. Also, this comment shows how the FC responded to such expectations by setting limits:

“Receiving orders from my husband was a problem. If I had learned everything beforehand, I would have been able to do it right and be sure of myself. It was an annoyance to begin with, that I did wrong. And still he was dependent on my help. But then I said *do you know what, this should be equal”.* (FC9)

Struggling in the role as caregiver was recognizable to other FCs as well. One FC found it difficult to set boundaries for her role and found it hard to tell other family members about how she struggled in her role. She held back her feelings for fear of being perceived as negative or being the one who prevented the family member from staying at home:

“I felt guilty (.) because I must be kind and decent. It could be a pressure to be a family caregiver”. (FC6)

The family relationship was also positively influenced during and after the caregiver experience in HaH. Several patients and FCs reported that the relationship between them had grown stronger due to the experiences of managing new roles and challenges together. Patients and FCs described a feeling of being proud of themselves and their partner. As one patient told, she was impressed by her husband and how well he handled the situation:

“He was really good at the pump, and he found solutions all the time. I found out that he could have been working as a nurse”. (P6)

One FC reflected how the family bond became stronger because of the shared experience in managing a serious life event:

“It was important for the children also, that he came home and that they were involved. They had not seen him for a long time, and when he came home, they could see him instead of imagining how ill he was. And they were so helpful to him. It kind of saved us, regarding psyche and family life and to keep our heads up”. (FC5)

## FCs role gradually diminishes and looking back

### A smooth transition to life beyond hospital at home

FCs and patients commonly described a smooth transition from stay in HaH to the previous well-known everyday life. FCs tasks decreased when the patients’ need for support diminished as the end of the patient treatment in HaH came closer. FCs and patients did rarely specifically mention the HaH treatment closure.

Patients became more independent when their infection was treated, and their health improved. One patient said she recovered well after she came home and that her need for care gradually diminished:

“The strange thing is that when I got home, the recovery process soon started. I became more active and slept better, and I simply felt better. Gradually I managed more on my own and was less dependent of my husband”. (P1)

FCs confirmed the recovery of patients after homecoming, contributing to a more normal life. One described:

“My husband recovered when he got home. As his energy returned, everything went back to normal again”. (FC5)

Furthermore, medical technical procedures and other tasks gradually became a routine, which also contributed to normalize FCs and the families everyday life. As one FC put it:

“The children soon got used to the sounds from the pump and understood that it was nothing to be afraid of. And they saw that he recovered (.) then the family got going again. The next month, everyday life was as before “. (FC7)

#### Finding motivation and meaning in providing care

Overall, the FCs mainly perceived HaH as a good choice for themselves, the patients, and their families. They all agreed that they would take up the role as a caregiver again if needed. The patients also thought of HaH as a good solution, both for themselves and for their family, when recalling their HaH experience.

One patient described she felt that her husband found it meaningful to support and care for her during the HaH treatment although he was not involved in the decision for choosing HaH in the initial phase:

“I think he felt it was a natural thing to do. To help me during the treatment at home. We had always helped each other. I think he found it enriching (.) when he looked back. Although he was never asked if it was okay in the first place and sometimes difficult”. (P1)

FCs confirmed this altruistic attitude in which they wanted to fulfil their family member’s wish to return home. By doing so, FCs experienced that they reduced their loved one’s distress due to hospitalization and thus, improved their quality of life and recovery. FCs mentioned that they found it meaningful to do so, even if it meant putting the patient’s needs before their own. One FC described:

“I knew him so well that I knew he would be getting healthier when coming home, quite simply. So (.) it was his choice, but I was in. I did not want to stress him out, but to build him up again. You set yourself aside a little. I have to say that. That is how it is (.) it is all the others in the family that matter, and you should be the strong one”. (FC5)

The FCs also described self-serving motivations driven by internal desires. They embraced the chance to be together with their loved one again. Another important aspect brought up by the FCs in this study was to avoid stress due to travelling between hospital and home and having obligations at both places. One FC reflected:

“I thought, if we were to travel to the hospital many times a day in several weeks (.) then we would have to reconsider our job situation. It would have interfered our everyday life, we have children at home too, right? So, hospitalization would affect us even more”. (FC5)

Another FC commented:

“Clearly my everyday life was affected, but less than when she was in the hospital. Because, in the hospital there were so many time limits. And she has been so many days in hospital over the years. Its a strain on the family as well, not at least leaving the hospital. When she came home it was easier for me, also mentally. I could see what was going on”. (FC4)

The two latter comments illustrate that FCs had an internal desire towards a calmer and more normal life, compared to hospitalization. In addition, emotions like love and affection seem to be a motivator as well.

## Discussion

The findings in this study show how the experiences of FC involvement and role in HaH were changing across different phases during HaH treatment. Even if previous HaH studies have not elaborated on FC involvement and role over time, some have pointed to the none-static nature of FC engagement [30, 34]. Our finding of a dynamic FC role is in accordance with research on family caregiving in various care environments, stating that FC involvement reflects the course of the patient’s illness and treatment whose different phases entail different needs for care [26, 27]. Identifying phases of FC involvement allows for a deeper understanding of the dynamic nature of the caregiving experience across the care continuum [27].

The course of family caregiving in HaH for adults, as it appears in the current study, is characterized by a sudden and intense onset in the initial phase, before the FCs adjust to the new role in the intermediate phase at home. Then, in line with the patients decreasing need for care, the FCs involvement, effort and stress gradually diminished in the final phase. Thus, the course of caregiving in HaH can be viewed as reversed to caregiver involvement in chronic illness, which is associated with an invisible and gradual onset and patients’ progressive needs for care [26, 44, 45].

The current study found that FCs seemed to be little prepared for the caregiver role in HaH. This finding is supported by Rossinot et al. [30], who showed that FCs were not adequately prepared for the role in the sense that they understood what HaH entailed. Thus, FCs in HaH can be equated with FCs in other caregiving contexts, being insufficiently prepared for the caregiver role due to lack of knowledge and skills required to provide care [46, 47]. FCs in the current study reported variable and mostly unmet informational needs and skills, despite the fact that they were met by HHPs who were eager to provide information and training. Both the patients and FCs perceived information and training as comprehensive and overwhelming, and they found it difficult to sort out the essential information from information that was not essential. Ones consider this in the context of information overload [48], which is linked to negative health outcomes for FCs [49]. Thus, HHPs should develop interventions to ensure more adequate and targeted information and training, to better prepare FCs for the caregiver role, tasks and responsibilities in HaH [50].

A finding in this study was that the FCs were excluded from the decision-making process of choosing HaH as a treatment option. The decision was mostly made by the patients and the hospital physicians. This result corroborates the findings of a meta-synthesis by Chua et al. [33]. Some have stated that patients and FCs prefer FC involvement in treatment decision making [51, 52] and that lack of FC involvement is linked to adverse consequences [33, 51]. Our finding is more nuanced. FCs in the current study did not question their omission from the decision of choosing HaH in the initial phase. They expressed an altruistic attitude and felt it was a natural thing for them to support and respect the patient’s choice for HaH. The FCs also had self-serving motivations driven by internal desires for the patients to come home. Also, when recalling their caregiver experience in HaH, FCs were commonly satisfied, and they perceived HaH treatment to be a good choice for both the patients and them. However, a few FCs in our study reported that they found it difficult to set boundaries for the caregiver role and they perceived it was hard to tell others that they were struggling in their role. This finding highlights an issue discussed in earlier research, whether the eagerness of patients to return home may lead to FCs feeling pressured to involve themselves as caregivers in HaH [28]. Such external pressure to provide care increases the likelihood of negative impact on the FCs [53, 54], and a better solution may be to encourage HHPs to invite FCs to play an active role if it is in line with the patient’s wishes [55].

Another finding in our study was the emphasis of coherent care and support from competent MHP during HaH. FCs embraced the continuity and stability within the MHP care team, and the high quality of patient care provided by these professionals were leading to feelings of trust and shared responsibility in FCs. Regular home visits, good availability, precise communication with MHPs and being connected with a municipal response center 24/7 were highlighted as supportive elements for FCs during their caregiver experience in HaH. This is in line with a study of Ko et al. [31] underlining the important role of a dedicated and competent HaH care team providing continuity of care in the patient home, supporting FCs with trust and reassurance to be the ones standing by the patient 24 hours a day. Still, in the study of Ko et al. [31] the HaH care team consisted of HHP, supported by private healthcare providers. Therefore, it seems that the composition of the HaH care team is not necessarily decisive for FC satisfaction and experience of support. More importantly for the FCs seemed to be the stability within the HaH care team and the trust in reaching out to them for help at any time. The finding of stability within the HaH care team as an enabler is consistent with that of Montalto [28] who suggested that patients and FCs are in need of a consistent approach, which is best carried out with a small pool of healthcare professionals.

Our results add to the limited body of research regarding the importance of relational aspects of providing care in HaH. Pre-established roles and characteristics of the family members seemed to influence the experiences of FC involvement and role in the current study. Patients with a healthcare professional background initially overestimated their own capacity, and HHPs and FCs let them do so. In such cases FCs reported to be less prepared for their tasks, effort, and responsibility in HaH. Thus, these FCs experienced a high level of distress. This should have warranted more attention and support from the HHPs. Our finding is in line with Svantesson et al. [56] who show that the tendency to overestimate capacity to cope in patients with professional healthcare background might lead to insecure care. Patients with healthcare background should be cared for just as any other patient. Furthermore, Prenkert et al. [57] advocate person-centered care, which promotes a holistic approach, including not only the patient but also the patient’s family [58]. Such approach can better tailor support of the unique needs of both patients and FCs in future HaH treatment [59].

The relationships between the patients and the FCs in our study were affected by the caregiving demand in HaH. Some patients and FCs described a tension in the relationship between them related to different views on the limitations of the FCs role and responsibilities. On the other hand, such tensions did not lead to conflicts and were temporary in nature. Caregiving in HaH also led to positive outcomes for the relationship between patients and FCs in this study, such as developing stronger bond and feeling proud of each other when struggling and finding solutions together. The FCs and patients in our study seemed to work together to regain a balanced and functional family system. Thus these findings can be considered within the Circumplex model, hypothesizing that balanced family systems are more functional than unbalanced family systems [60]. The unbalance in the interpersonal relationship which arises when one family member becomes sick, puts the family in need for re-construction to achieve balance. FCs and patients in our study seemed to work together to regain such a balance. Our findings are supported by Makela et al. [34] who found that patients and FCs were working closely together in new ways to adapt to the new situation. Furthermore, Rossinot et al. [30] demonstrated how caregiving in HaH led to hard feelings like stress and guilt in both patients and FCs, which led to harm in the relationship. Still, our findings highlight that they are both benefits and costs for the relationship.

### Strengths and limitations

This study is one out of few studies reporting FC involvement and role when adult patients are treated and cared for in HaH, and to our knowledge, this is the first study to elaborate on the topic as it was perceived by both patients and FCs. Patients and FCs often live and interact closely together; thus, we found that the patients had valuable insight in FC involvement and role as well. We had no intention to compare the experiences from patients and FCs in this study, but we considered that the patients could enrich our understanding of the FC involvement and role. Another strength of this study was the variation in participants’ age, gender, diagnosis, type of family relationships and length of stay in HaH. A sample with such a broad range of characteristics was appropriate to capture different aspects of the phenomenon [38]. Involvement of the FC representative in the study and an analysis process involving all authors is a strength and contributed to a thorough understanding of the patients and the FCs descriptions.

As for the limitations of the study, there is a possibility that some aspects of the patients’ and FCs’ experiences may have been difficult to convey, as some time had passed since they were admitted to HaH. Also, one of the telephone-interviews was short, but included since it gave valuable information. Furthermore, a contextual factor should be discussed when interpreting our findings. Most of the study participants had experienced HaH during the time of the COVID-19 pandemic. Due to strict visiting regulations at the hospital at the time of the pandemic, most patients and FCs experienced being separated during the in-hospital stay. This separation was described as a negative experience which affected patients’ eagerness to choose HaH, as well as FCs wanting the patients to come home. Therefore, the pandemic situation could also have influenced the participants’ positive predictive outlook as well as their overall satisfaction with HaH. Also, the strict visit regulations in the time of the COVID-19 pandemic could have contributed to FCs being left out of the decision process, as this decision was taken while the patient was hospitalized.

The findings reflect the experiences and descriptions of patients and FCs for a specific area and HaH model in Norway. Thus, it is possible the transfer value of the findings may be restricted to comparable HaH settings and complexities of treatment.

## Conclusion

The FCs in this study played an important role in HaH, although their tasks, involvement and effort varied across different phases during HaH treatment. The findings contribute to a greater understanding of the dynamic nature of the caregiver experiences during HaH treatment, which can guide healthcare professionals on how to time appropriate support to FCs in HaH when they are most in need. Such knowledge is important to decrease the risk of caregiver distress during HaH treatment. Further work, such as longitudinal studies, should be done to examine the course of caregiving in HaH over time to correct or support the phases described in this study.

## Electronic supplementary material

Below is the link to the electronic supplementary material.

Supplementary Material 1

Supplementary Material 2

Supplementary Material 3

## Data Availability

The datasets generated and analyzed during the current study are not publicly available due to the Norwegian law regarding confidentiality and privacy of participants. If desired, selected, de-identified quotes are available from the corresponding author (LK) on reasonable request. The interview guide is also available upon request from the corresponding author.

## List of abbreviations

HaH: Hospital at home
FC: Family caregiver
HHP: Hospital healthcare professional
MHP: Municipal healthcare professional
