# Supplementary material for: Family caregiver involvement and role in hospital at home for adults: the patients’ and family caregivers’ perspective - a Norwegian qualitative study
Source: BMC Health Serv Res. 2023 May 17;23:499. doi: 10.1186/s12913-023-09531-3 (PMC10189695; doi:10.1186/s12913-023-09531-3)
Supplement: Supplementary file 1 — Supplementary Material 1 [file 12913_2023_9531_MOESM1_ESM.pdf]

Interview guide

1. How did you experience *you as a family caregiver/ your family caregiver* becoming involved in the HaH?

2. Did you experience that *you as a family caregiver/ your family* caregiver were given any tasks in HaH?

3. Did you experience that *you as a family caregiver/ your family caregiver* had any form of responsibility in HaH?

4. How did you experience that *you as a caregiver/ your family caregiver* were cared for by healthcare personnel and how was the cooperation and communication with these professionals?

5. Did *you as a family caregiver/your family caregiver* experience any challenges during the process, and if so which ones?

6. Did *you as a family caregiver/ your family caregiver* have any positive experiences along the way?

7. Is there anything else you would like to add? Something we haven't talked about?
