# Supplementary material for: Family caregiver involvement and role in hospital at home for adults: the patients’ and family caregivers’ perspective - a Norwegian qualitative study
Source: BMC Health Serv Res. 2023 May 17;23:499. doi: 10.1186/s12913-023-09531-3 (PMC10189695; doi:10.1186/s12913-023-09531-3)
Supplement: Supplementary file 3 — Supplementary Material 3 [file 12913_2023_9531_MOESM3_ESM.pdf]

Supplementary material file legends:

Additional file 1, PDF, Interview guide

Additional file 2, PDF, Consolidated criteria for reporting qualitative studies (COREQ): 32-item checklist
